# Two strains of *Bacillus velezensis*: potent probiotics against *Escherichia coli* K99-induced diarrhea with high safety in mice

**DOI:** 10.1128/spectrum.01214-25

**Published:** 2025-10-20

**Authors:** Fengjie Wang, Yanan Wang, Hongjun Zhang, Bowei Zhao, Jian Li, Ruili Shi, Li Chen, Zhiqiang Zhang, Qiumei Shi, Qinghui Jia, Tonglei Wu

**Affiliations:** 1 Hebei Provincial Key Laboratory of Preventive Veterinary Medicine, Hebei Normal University of Science and Technology 165079, Qinhuangdao, PR China; 2 Chengde City Academy of Agricultural and Forestry Sciences, Chengde, PR China; 3 Weichang Manchu and Mongolian Autonomous County Animal Epidemic Control Center, Chengde, PR China; 4 Hebei Provincial Animal Husbandry General Station, Shijiazhuang, PR China; 5 Hebei Tourism Vocational College, Chengde, PR China; Nanjing Agricultural University, Nanjing, China

**Keywords:** *Bacillus*, *Escherichia coli *K99, diarrhea, mice, probiotics

## Abstract

*Escherichia coli* K99 is a primary causative agent of diarrhea in young animals, leading to significant health and economic losses. With growing concerns over antibiotic resistance, probiotics—particularly *Bacillus* species—are gaining attention as effective alternatives for disease prevention and health promotion in livestock. In this study, 39 *Bacillus* strains were isolated from fresh cattle feces. Preliminary screening focused on biofilm formation, hemolytic activity, and antibacterial effects against *E. coli* K99. Promising candidates were further evaluated for *in vitro* probiotic characteristics and safety, including tolerance to acidic and bile salt conditions, heat resistance, hydrophobicity, enzymatic activity, and the presence of virulence and antibiotic resistance genes. *In vivo* assessments involved acute and subchronic toxicity tests and protective efficacy evaluations in a mouse model challenged with *E. coli* K99. Four strains—T36, B102, B116, and B150—demonstrated strong biofilm-forming ability, non-hemolytic properties, and notable antimicrobial activity. Among them, B102 and B116 showed excellent probiotic traits and safety profiles *in vitro*, lacking gelatinase and lecithinase activity, and were free of detectable virulence or resistance genes. *In vivo*, oral administration of B102 and B116 at high doses caused no acute or subchronic toxicity in mice. Notably, these strains improved the survival rate of *E. coli* K99-infected mice to 50% and 30%, respectively, compared to 0% in the untreated group. In conclusion, strains B102 and B116 exhibit strong probiotic potential and safety, offering promising alternatives to antibiotics for the prevention and control of *E. coli* K99 infections in animals. Their application may contribute to sustainable livestock health management.

**IMPORTANCE:**

*Escherichia coli* K99-induced diarrhea causes severe economic losses in livestock farming, with limited safe control options due to antibiotic resistance. This study identifies two *Bacillus velezensis* strains (B102 and B116) that effectively inhibit *E. coli* K99, enhance survival rates in infected mice, and exhibit strong safety profiles. Their tolerance to gastrointestinal stress and lack of virulence or resistance genes make them ideal antibiotic alternatives. These strains could promote sustainable livestock health by reducing antibiotic use, mitigating resistance risks, and improving disease control, benefiting both agricultural productivity and public health.

## INTRODUCTION

*Escherichia coli* is among the most adaptable and pathogenic bacteria, responsible for a wide range of infectious diseases in animals, particularly gastrointestinal and systemic infections. It poses a significant threat to the intestinal health of livestock and poultry (1–3). *E. coli* K99 is a well-documented serotype known for its strong adhesion capability and toxin production, which trigger inflammatory responses and disrupt intestinal tight junctions, ultimately leading to diarrhea in piglets and calves (3–5). Antibiotics remain a primary strategy for controlling *E. coli* K99 infections; however, their excessive use leads to antibiotic residues in animal-derived food products, posing a threat to food safety. Moreover, it contributes to environmental pollution and the emergence of multidrug-resistant strains, which present additional risks to human health (4). A healthy gut microbiota prevents colonization of foreign pathogens and enhances host immune systems through interactions between antigens and immune cells during early stages of life (6). Probiotics have emerged as a promising solution due to their ability to regulate intestinal microbiota and inhibit pathogenic bacterial colonization (7). Studies indicate that early interventions, such as probiotic supplementation, fecal microbiota transplantation, and rumen microbiota transplantation, can effectively mitigate calf diarrhea (8). Probiotics help maintain intestinal homeostasis, enhance immunity, and reduce the risk of *E. coli* K99 infections, making them a viable strategy for minimizing antibiotic use and improving overall animal health (9).

Among various probiotic strains, *Bacillus* species are widely recognized as functional feed additives (10, 11). These gram-positive, aerobic bacteria can form resilient spores that withstand extreme conditions, including high temperatures, pH variations, dehydration, and environmental stressors (12). *Bacillus* exhibits unique biological functions, such as promoting digestion, enhancing immunity, and exerting antimicrobial and disease-preventive effects. With growing research and interest in antibiotic-free farming and functional food development, *Bacillus* has demonstrated considerable potential in animal health management (13).

Extensive studies highlight *Bacillus*’ ability to produce antimicrobial compounds, extracellular digestive enzymes, antimicrobial peptides, and other bioactive molecules. Furthermore, *Bacillus* actively modulates host immune responses, enhances immune cell activity, and contributes to pathogen resistance, reducing inflammation and even lowering blood lipid levels (14). Due to its natural and safe properties, *Bacillus* is widely used in the breeding and production of poultry, swine, and ruminants (15). It improves feed digestion, nutrient utilization, and overall animal health while significantly reducing antibiotic dependence (16, 17). Given the global push for antibiotic-free livestock production, *Bacillus* represents a valuable alternative for maintaining intestinal health and enhancing immune function, supporting the development of a sustainable and healthy livestock industry.

However, while most *Bacillus* strains exhibit beneficial properties, some may act as opportunistic pathogens under specific conditions. Certain strains, such as *Bacillus cereus*, can carry virulence factors like hemolysin and gelatinase, compromising intestinal integrity and facilitating pathogen invasion (18). Some *Bacillus* strains produce enterotoxins that can lead to foodborne illnesses, primarily characterized by diarrhea and vomiting (16). Moreover, excessive *Bacillus* supplementation may cause biochemical imbalances and organ damage, particularly affecting the liver. Certain *Bacillus* species can also translocate to other organs, potentially causing localized or systemic infections. Therefore, rigorous safety assessments are essential before introducing a new probiotic strain into practical applications.

In this study, *Bacillus* strains were isolated from fresh cow feces. To comprehensively evaluate their probiotic potential and safety, we conducted systematic *in vitro* and *in vivo* experiments, including a challenge-protection study using a mouse diarrhea model. The findings provide a scientific basis for the application of *Bacillus* in preventing and controlling *E. coli* K99-induced diarrheal diseases in animals.

## MATERIALS AND METHODS

### Strains and cells

*Bacillus* strains were preserved at the Key Laboratory of Veterinary Preventive Medicine of Hebei Province. *Bacillus subtilis* SHBCC D50761 was obtained from the Shanghai Bioresource Collection Center. *Pseudomonas aeruginosa* ATCC 9027 was obtained from the Shanghai Bioresource Collection Center. *E. coli* K99 was isolated from a beef cattle farm. All strains were cultured in Brain Heart Infusion (BHI) broth liquid medium and on BHI agar plates at 37°C.

### Experimental animal

In accordance with the standards outlined in GB 15193.22-2014, GB 15193.3-2014, and GB 14925-2023, female Kunming (KM) mice, aged 6 to 8 weeks, were used as experimental subjects. The animals were housed in a sterile environment under standardized conditions, including a controlled temperature of 22.0°C (±0.5°C), a relative humidity of 60% (±10%), and a 12 hour light/dark cycle. All mice were procured from Spay (Beijing) Biotechnology Co., Ltd., China.

### Isolation and identification of *Bacillus* strains

Two grams of fresh feces were mixed with 10 mL of sterile phosphate-buffered saline (PBS), and 100 µL of the resulting suspension was spread onto BHI agar plates. The plates were incubated at 37°C overnight. Colony morphology, color, and size were observed, and suspected *Bacillus* colonies with crater-like centers and wrinkled edges were selected using an inoculation loop. These colonies were further purified to obtain single isolates.

Physiological and biochemical characteristics of the isolates were determined using bacterial microbiochemical identification tubes (Qingdao High-Tech Industrial Park Haibo Biotechnology Co., Qingdao, China). Briefly, single bacterial colonies were suspended in sterile saline to achieve a turbidity of 0.5 McFarland standard. Then, 50 µL–100 µL of the bacterial suspension was inoculated into the biochemical identification tubes and incubated at 37°C for the time specified in the manufacturer’s instructions. The results were interpreted according to Bergey’s Manual of Systematic Bacteriology.

Genomic DNA was extracted from the isolates using a bacterial genome extraction kit (Tiangen, China) following the manufacturer’s instructions. The 16S rRNA gene was amplified by PCR using the primers F (5′-AGAGTTTGATCCTGGCTCAG-3′) and R (5′-GGTTACCTTGTTACGACTT-3′). The resulting PCR products were sent to Sangon Biotech (Shanghai) Co., Ltd. for sequencing. The obtained sequences were subjected to BLAST analysis for further identification of *Bacillus* species.

### Assessment of biofilm formation ability

The ability of the isolates to form biofilms was assessed using the crystal violet staining method, as described in the reference (19). A single bacterial colony was inoculated into BHI liquid medium and incubated aerobically at 37°C overnight. The overnight culture was then diluted 1:100 in fresh BHI liquid medium, and 200 µL of the diluted suspension was added to each well of a 96-well microtiter plate. The plate was incubated statically at 37°C for 36 hours.

Following incubation, the culture medium was discarded, and the wells were gently washed two times with PBS. The biofilms were then fixed with 200 µL of absolute methanol for 15 min, followed by another two gentle washes with PBS. Next, 200 µL of a 2% crystal violet solution (prepared in deionized water) was added to each well for staining, and the plate was incubated for 15 min. Excess crystal violet was removed, and the wells were gently washed with deionized water. The microtiter plate was then inverted on filter paper to remove residual moisture.

To solubilize the bound crystal violet, 200 µL of 33% glacial acetic acid was added to each well, and the plate was incubated for 30 min. The absorbance of the solution was measured at 595 nm (OD_595_). Each experiment was performed in triplicate, and the average absorbance (D) was calculated. An uninoculated medium was used as a negative control, and twice the absorbance of the negative control (Dc) was set as the threshold. Biofilm formation was categorized as follows: strong (D > 2 × Dc), weak (Dc < D ≤ 2 × Dc), or absent (D ≤ Dc).

### Antimicrobial activity analysis

*E. coli* K99 at the logarithmic phase of growth was adjusted to a concentration of 1 × 10^7^ colony-forming unit (CFU)/mL, and 200 µL of this suspension was evenly spread on BHI agar plates. A single colony of strains was inoculated into BHI liquid medium and incubated aerobically at 37°C overnight. The next day, 5 µL of the culture was spotted onto agar plates coated with *E. coli* K99, followed by incubation aerobically at 37°C for 12 hours. The diameter of the inhibition zone was measured and recorded. Similarly, *B. subtilis* SHBCC D50761 was prepared as a positive control, and PBS was used as a negative control.

### Hemolytic activity assay

A single colony of strains was inoculated into BHI liquid medium and incubated aerobically at 37°C overnight. The next day, 5 µL of the culture was spotted onto blood agar plates, followed by incubation aerobically at 37°C for 24 hours. The hemolytic reaction was evaluated based on the presence of a clear area around the bacterial colonies (total hemolysis, β-hemolysis), a green zone (partial hemolysis, α-hemolysis), or no reaction (γ-hemolysis). The assay was performed in triplicate and on two independent occasions. *Staphylococcus aureus* CMCC(B)26003 was used as the positive control for hemolytic activity.

### Analysis of probiotic properties of strains *in vitro*

#### Hydrophobicity, autoaggregation, and coaggregation assays

The surface hydrophobicity of bacterial strains was evaluated using the xylene assay. The experimental procedure was as follows: the bacterial strains were inoculated into BHI liquid medium and incubated aerobically at 37°C overnight. Following incubation, the cultures were centrifuged at 8,000 rpm for 15 min at 4°C, and the supernatant was discarded. The bacterial pellets were then washed twice with PBS and resuspended, adjusting the optical density at 600 nm (OD_600_) to 0.6. *B. subtilis* SHBCC D50761 was used as a positive control in all assays.

##### Surface hydrophobicity assay

To assess bacterial surface hydrophobicity, 1 mL of xylene was added to 3 mL of bacterial suspension (OD_600_ = 0.6) in a centrifuge tube. The mixture was vortexed thoroughly and allowed to stand at room temperature for 1 hour until phase separation occurred. The OD_600_ of the aqueous phase was then measured. A control group was prepared by mixing 1 mL of xylene with 3 mL of PBS. The surface hydrophobicity rate (%) was calculated using the following formula: surface hydrophobicity rate (%) = (A_0_ – A_1_) / A_0_ × 100%, where A_0_ represents the OD_600_ of the bacterial suspension before mixing, and A_1_ represents the OD_600_ of the aqueous phase after 1 hour.

##### Autoaggregation assay

For the autoaggregation analysis, 5 mL of bacterial suspension (OD_600_ = 0.6) was incubated at room temperature for 2 hours. Subsequently, 3 mL of the upper phase was carefully removed, and its OD_600_ was recorded. The autoaggregation rate (%) was determined using the following formula: autoaggregation rate (%) =(A_0_ – A_2_) / A_0_ × 100%, where A_0_ is the OD_600_ of the bacterial suspension immediately after mixing, and A_2_ is the OD_600_ after 2 hours of static incubation.

##### Coaggregation assay

To evaluate coaggregation, *E. coli* K99 was inoculated into BHI liquid medium and incubated overnight at 37°C. After incubation, the cultures were centrifuged at 8,000 rpm for 15 min at 4°C, and the supernatant was discarded. The bacterial pellets were washed with PBS and resuspended to an OD_600_ of 0.6. Equal volumes (2 mL each) of *E. coli* K99 were combined with 2 mL of T36, B102, B116, and B150 suspensions (OD_600_ = 0.6). The mixtures were incubated at room temperature for 2 hours. After incubation, 3 mL of the upper phase was removed, and the OD_600_ was recorded. *B. subtilis* SHBCC D50761 was used as the positive control. The coaggregation rate (%) was calculated using the following equation: coaggregation rate (%) = (A_0_ – A_3_) / A_0_ × 100%, where A_0_ represents the OD_600_ of the mixed bacterial suspension immediately after mixing, and A_3_ represents the OD_600_ after 2 hours of incubation.

These assays provide critical insights into the adhesion properties of the bacterial strains, which may have implications for their potential applications in microbial ecology and probiotic research.

### Acid, bile salt, and heat resistance assays

Tolerance to acidic environments, bile salts, and elevated temperatures is a fundamental prerequisite for the probiotic efficacy of bacterial strains. The following assays were conducted to assess the resilience of T36, B102, B116, and B150 under these stress conditions, with *B. subtilis* SHBCC D50761 serving as the positive control.

#### Acid tolerance assay

To evaluate acid tolerance, overnight cultures of T36, B102, B116, and B150 were prepared by inoculating each strain into BHI liquid medium and incubating aerobically at 37°C. The following day, a 1:50 dilution (1 mL) of the overnight culture was introduced into BHI medium adjusted to pH 2, pH 3, pH 4, and pH 5. The cultures were incubated aerobically at 37°C for 4 hours. Bacterial viability was assessed at 0 and 4 hours using the drop plate method.

#### Bile salt tolerance assay

For bile salt tolerance assessment, overnight cultures of T36, B102, B116, and B150 were diluted 1:50 (1 mL) and inoculated into BHI liquid medium supplemented with bile salts at concentrations of 0.1%, 0.2%, and 0.3% (wt/vol). The cultures were incubated aerobically at 37°C for 4 hours, and bacterial counts were determined at 0 and 4 hours using the drop plate method.

#### Heat resistance assay

To determine heat resistance, overnight cultures of T36, B102, B116, and B150 were diluted 1:50 (1 mL) and transferred into 50 mL of fresh BHI medium. The cultures were incubated aerobically at 37°C for 4 hours before being subjected to thermal stress in a thermostatic water bath at 60°C, 70°C, and 80°C for 15 min. Bacterial survival was quantified at each temperature point using the drop plate method.

These assessments provide valuable insights into the robustness of the bacterial strains under gastrointestinal and environmental stress conditions, contributing to their potential applications in probiotic development.

### Assessment of tolerance to simulated gastric and intestinal fluids

Simulated gastric and intestinal fluids were prepared by adjusting BHI liquid medium to pH 3 and pH 8, respectively. The media were sterilized by autoclaving and subsequently cooled to room temperature. Pepsin (1 g/L) was added to the simulated gastric fluid, while trypsin (1 g/L, 1:250) was incorporated into the simulated intestinal fluid. The prepared solutions were stored at 4°C until use.

A single colony of T36, B102, B116, and B150 was inoculated into BHI liquid medium and incubated aerobically at 37°C overnight. The following day, a 1:50 dilution (1 mL) of the overnight culture was transferred into the prepared simulated gastric and intestinal fluids and incubated aerobically at 37°C for 4 hours. Bacterial viability was assessed at 0 and 4 hours using the drop plate method. *B. subtilis* SHBCC D50761 served as the positive control.

These assays provide critical insights into the resilience of the bacterial strains under gastrointestinal conditions, contributing to their potential application as probiotics.

### *In vitro* safety assessment of T36, B102, B116, and B150

#### Gelatinase activity assay

To evaluate gelatinase activity, a single colony of T36, B102, B116, and B150 was inoculated into BHI liquid medium and incubated aerobically at 37°C overnight. The following day, 5 µL of the overnight culture was spotted onto gelatin agar plates and incubated aerobically at 37°C for 24 hours. The formation of clear zones around the colonies was examined to indicate gelatin hydrolysis. *P. aeruginosa* ATCC 9027 served as the positive control for gelatinase activity.

#### Antibiotic susceptibility testing

The antibiotic resistance profiles of T36, B102, B116, and B150 were determined using the Kirby-Bauer disk diffusion method. A total of 24 antibiotic disks (Cat. No. S1100, Hangzhou Microbial Reagents Co., Hangzhou, China) were tested.

For each strain, 200 µL of the overnight culture was evenly spread onto BHI agar plates, and antibiotic disks were placed on the surface. The plates were then incubated aerobically at 37°C for 24 hours. The diameter of the inhibition zones was measured, and susceptibility was classified as sensitive (S), intermediate (I), or resistant (R) according to the guidelines set by the National Committee for Clinical Laboratory Standards.

#### PCR detection of antibiotic resistance and virulence genes

PCR amplification was conducted to detect antibiotic resistance and virulence genes in T36, B102, B116, and B150. Primers for antibiotic resistance genes and virulence genes were designed using Primer 5 software and synthesized by Sangon Biotech Co., Ltd. (Shanghai, China). The primer sequences and annealing temperatures used for amplification are detailed in Table 1. Genomic DNA extracted from each strain served as the template for PCR analysis. The reactions were performed in a 25 µL reaction mixture containing 0.4 µM of each primer (Table 1), 2 µL of template DNA, 12.5 µL of 2× Taq Master Mix (CwBio Co., Jiangsu, China), and nuclease-free water to a final volume of 25 µL.

**TABLE 1:** Primers used in the detection of antibiotic resistance and virulence genes

Primers	Sequence (5´−3´)	Product size (bp)	Annealing temperature (°C)
*vanA*-for	GGGAAAACGACAATTGC	732	53
*vanA*-rev	GTACAATGCGGCCGTTA		
*vanB*-for	GTGCTGCGAGATACCACAGA	635	53
*vanB*-rev	CGAACACCATGCAACATTTC		
*lnuA*-for	GGTGGCTGGGGGGTAGATGTATTAACTGG	323	61
*lnuA*-rev	GCTTCTTTTGAAATACATGGTATTTTTCGATC		
*rpoB*-for	CCCAGGACGTGGAGGCGATCAC	365	54
*rpoB*-rev	TGCCGCACCAATCGCTGCTC		
*hblA*-for	AAGCAATGGAATACAATGGG	1,154	56
*hblA*-rev	AGAATCTAAATCATGCCACTGC		
*hblC*-for	GATACCAATGTGGCAACTGC	740	58
*hblC*-rev	TTGAGACTGCTCGCTAGTTG		
*hblD*-for	ACCGGTAACACTATTCATGC	829	58
*hblD*-rev	GAGTCCATATGCTTAGATGC		
*nheA*-for	TACGCTAAGGAGGGGCA	499	56
*nheA*-rev	GTTTTTATTGCTTCATCGGCT		
*nheB*-for	CTATCAGCACTTATGGCAG	769	54
*nheB*-rev	ACTCCTAGCGGTGTTCC		
*nheC*-for	CGGTAGTGATTGCTGGG	581	58
*nheC*-rev	CAGCATTCGTACTTGCCAA		

The PCR conditions included an initial denaturation at 95°C for 10 min, followed by 30 cycles of denaturation at 95°C for 30 seconds, annealing at the corresponding temperature for 30 seconds, and extension at 72°C for 30 seconds, with a final extension step at 72°C for 5 min. The PCR products were analyzed via electrophoresis on a 1% agarose gel.

These safety evaluations provide essential insights into the potential probiotic applications of T36, B102, B116, and B150 by assessing their enzymatic activity, antibiotic resistance, and genetic determinants associated with virulence.

### *In vivo* safety evaluation of T36, B102, B116, and B150

#### Acute oral toxicity assessment

The acute oral toxicity study was conducted in accordance with the guidelines outlined in GB 15193.3-2014, “Acute Oral Toxicity Test.” A total of 20 female Kunming mice (6–8 weeks old) were randomly assigned to four experimental groups (*n* = 5 per group) (20). Group A received an oral dose of B102 at 2 × 10⁹ CFU per mouse, group B was administered B116 at the same dosage, and group C received *B. subtilis* SHBCC D50761 at 2 × 10⁹ CFU per mouse. Group D, serving as the control, was given 200 µL of PBS. The animals underwent continuous treatment for 14 days, during which their clinical symptoms were closely monitored and systematically recorded on a daily basis.

#### 28-Day repeated oral toxicity evaluation

The subchronic toxicity study was performed in compliance with GB 15193.22-2014, “28 Day Oral Toxicity Test.” A cohort of 40 female Kunming mice (6–8 weeks old) was randomly allocated into four experimental groups (*n* = 10 per group) (21). Group A received an oral dose of B102 at 2 × 10⁷ CFU per mouse, group B was administered B116 at the same concentration, and group C was treated with *B. subtilis* SHBCC D50761 at 2 × 10⁷ CFU per mouse. Group D served as the control and received 200 µL of PBS. The treatment regimen spanned 28 consecutive days. To comprehensively assess the safety profile of the probiotics, multiple parameters were evaluated, including body mass index (BMI), organ index, bacterial translocation, bacterial infection analysis, blood biochemical indices, and histopathological examinations.

##### BMI measurement

During the 28 day oral gavage experiment, BMI was measured at five specific time points: day 0, day 7, day 14, day 21, and day 28.

##### Organ index measurement

Following euthanasia, the heart, liver, spleen, lungs, and kidneys were aseptically harvested. Each organ was weighed, and the organ index was calculated using the following formula: organ index (%) = organ weight (g) / body weight (g) × 100

##### Probiotic translocation and infection analysis

After euthanasia, the heart, liver, spleen, lungs, and kidneys were collected under sterile conditions. Tissue samples from each organ were streaked onto BHI agar plates and incubated anaerobically at 37°C for 24 hours. The presence of bacterial colony growth was examined to assess probiotic translocation.

##### Blood biochemical analysis

Blood samples were collected from the tail vein, and serum was separated for further analysis. Various blood biochemical parameters, including liver and kidney function markers, blood glucose levels, lipid profiles, and cholesterol levels, were evaluated using an automatic biochemical analyzer (Jiangsu Su-Lian Ke Biological Technology Co., Jiangsu, China).

##### Histopathological analysis

Following euthanasia, 1 cm segments of the ileum were immediately fixed in pre-cooled 4% paraformaldehyde for 24 hours. The fixed tissues underwent graded ethanol dehydration (70%, 80%, 90%, 100%), followed by xylene infiltration and paraffin embedding. Thin sections (4 μm–6 μm) were prepared using a microtome and stained with hematoxylin and eosin. Histopathological changes were then examined under a light microscope.

### *In vivo* probiotic efficacy assessment

#### Cytokine quantification

Cytokine levels in serum, jejunum, and colon tissues were measured using a sandwich enzyme-linked immunosorbent assay (ELISA). Tissue samples (0.1 g) were homogenized in 1 mL of PBS and subsequently centrifuged at 3,000 rpm for 20 min at 4°C. The resulting supernatant was stored at −80°C for further analysis. Serum specimens were thawed prior to measurement. The concentrations of tumor necrosis factor-α (TNF-α), interleukin-6 (IL-6), and interleukin-10 (IL-10) in serum, jejunum, and colon tissues were determined using commercially available mouse ELISA kits (Jiangsu Su-Lian Ke Biological Technology Co., Jiangsu, China) following the manufacturer’s standard protocols.

#### Toxicity assessment of *E. coli* K99 and LD_50_ determination

The toxicity of *E. coli* K99 was evaluated in mice to establish the infectious dose. Thirty female Kunming mice (6–8 weeks old) were randomly assigned to six groups (*n* = 5 per group). The first five groups received intraperitoneal injections of *E. coli* K99 at doses of 5.56 × 10^8^ CFU per mouse, 5.56 × 10^7^ CFU per mouse, 5.56 × 10^6^ CFU per mouse, 5.56 × 10^5^ CFU per mouse, and 5.56 × 10^4^ CFU per mouse. The sixth group received an equal volume of PBS as a control. Mice were monitored for 14 days, and mortality was recorded. The median lethal dose (LD_50_) of *E. coli* K99 was calculated using the Spearman-Karber method: logLD_50_ = X_k_ − i (∑p − 0.5), where X_k_ is the highest logarithmic dose, i is the difference between two consecutive logarithmic doses, and ∑p is the total mortality rate across all groups.

#### Evaluation of the protective

To evaluate the protective effect of B102 and B116, 40 female Kunming mice (6–8 weeks old) were randomly assigned to four groups (*n* = 10). Group A mice received continuous oral administration of B102 (200 µL per mouse, 1 × 10^10^ CFU/mL) for 14 days, while mice in group B and group C were separately treated with B116 and *B. subtilis* SHBCC D50761 under the same dose. The mice from group D received 200 µL of PBS as the control. On day 14, all groups were intraperitoneally injected with *E. coli* K99 at a dose equivalent to 2 × LD_50_. The mice were monitored for an additional 14 days, during which morbidity and mortality were recorded.

### Statistical analysis

Statistical analysis was performed using IBM SPSS Statistics 26 software. A one-way analysis of variance was conducted, and *post hoc* comparisons between groups were performed using the least significant difference (LSD)-mean method. The Shapiro-Wilk test was used to assess the normality of the data between the two groups. After confirming the assumption of normal distribution, an independent samples *t*-test was performed. Data are presented as mean ± standard error of the mean. Statistically significant differences were indicated with asterisks (*), where *P < 0.05, **P < 0.01, and ***P < 0.001 denote significant differences in mean values, and ns indicates no significant difference.

## RESULTS

### Strain isolation and identification

Initially, 603 bacterial colonies were isolated from fecal samples and identified through morphological and biochemical tests. Colonies with a round shape, smooth edges, and white or pale-yellow color were selected for further purification. Gram staining was performed on the suspected strains, and the physiological and biochemical characteristics of the strains were determined using a bacterial microtiter plate biochemical identification kit. The results revealed that 39 suspected *Bacillus* strains tested positive for catalase, nitrate reduction, and the Voges-Proskauer reaction, while they were negative for indole, methyl red, and lecithinase tests. These strains were also capable of hydrolyzing glucose and starch. Based on the classification and identification standards from the Bergey’s Manual of Systematic Bacteriology, the 39 strains were identified as belonging to the genus *Bacillus*.

Furthermore, 16S rRNA gene sequencing and sequence alignment confirmed that the 39 isolated strains were primarily *Bacillus pumilus*, *Bacillus velezensis*, *Bacillus halodurans,* and *B. subtilis* (Table 2).

**TABLE 2:** The *Bacillus* species determined by 16S rRNA gene sequence comparison

Species	Species	Species	Species
B36: *B. pumilus*	B137: *B. safensis*	F140: *B. velezensis*	T48: *B. subtilis*
B43: *B. velezensis*	B139: *B. velezensis*	F144: *B. safensis*	T56: *B. altitudinis*
B53: *B. pumilus*	B142: *B. pumilus*	F173: *B. pumilus*	B46: *B. subtilis*
B60: *B. pumilus*	B150: *B. pumilus*	F179: *B. pumilus*	B65: *B. safensis*
B72: *B. subtilis*	B289: *B. cereus*	F186: *B. halodurans*	B126: *B. safensis*
B91: *B. pumilus*	F17: *B. velezensis*	F218: *B. velezensis*	F133: *B. halodurans*
B100: *B. pumilus*	F57: *B. pumilus*	T36: *B. halodurans*	F149: *B. velezensis*
B102: *B. velezensis*	F60: *B. velezensis*	T37: *B. halodurans*	F165: *B. altitudinis*
B114: *B. safensis*	F91: *B. velezensis*	T63: *B. halodurans*	F199: *B. subtilis*
B116: *B. velezensis*	F108: *B. velezensis*	T17: *B. subtilis*	

### *Bacillus* strains exhibited strong biofilm-forming abilities

**Fig 1:**
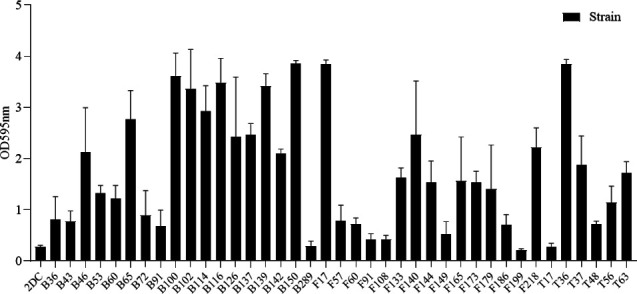
Determination of biofilm-forming capacity of 39 isolates. The data represent the average of three replicates. Bar mean with ±SD.

The biofilm formation of the *Bacillus* strains was assessed using the microplate quantification method. The results (Fig. 1) showed that OD_595_ for all 39 strains was significantly higher than 2 × Dc. Among these, *Bacillus* strains B150, F17, T36, B100, B116, B102, B139, and B114 had OD_595_ of 3.85, 3.84, 3.85, 3.61, 3.48, 3.36, 3.41, and 2.93, respectively, indicating that these eight strains exhibited particularly strong biofilm-forming capabilities.

### *Bacillus* strains exhibit antibacterial activity against *E. coli* K99

**Fig 2:**
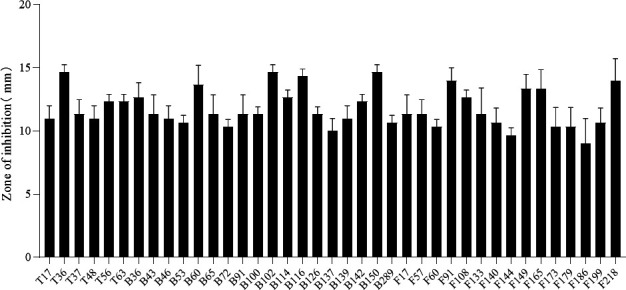
Inhibition results of 39 isolates against *E. coli* K99. Five microliters of the culture was spotted onto agar plates coated with *E. coli* K99, followed by incubation aerobically at 37°C for 12 hours. The diameter of the inhibition zone was measured and recorded. The data represent the average of three replicates. Bar mean with ±SD.

**Fig 3:**
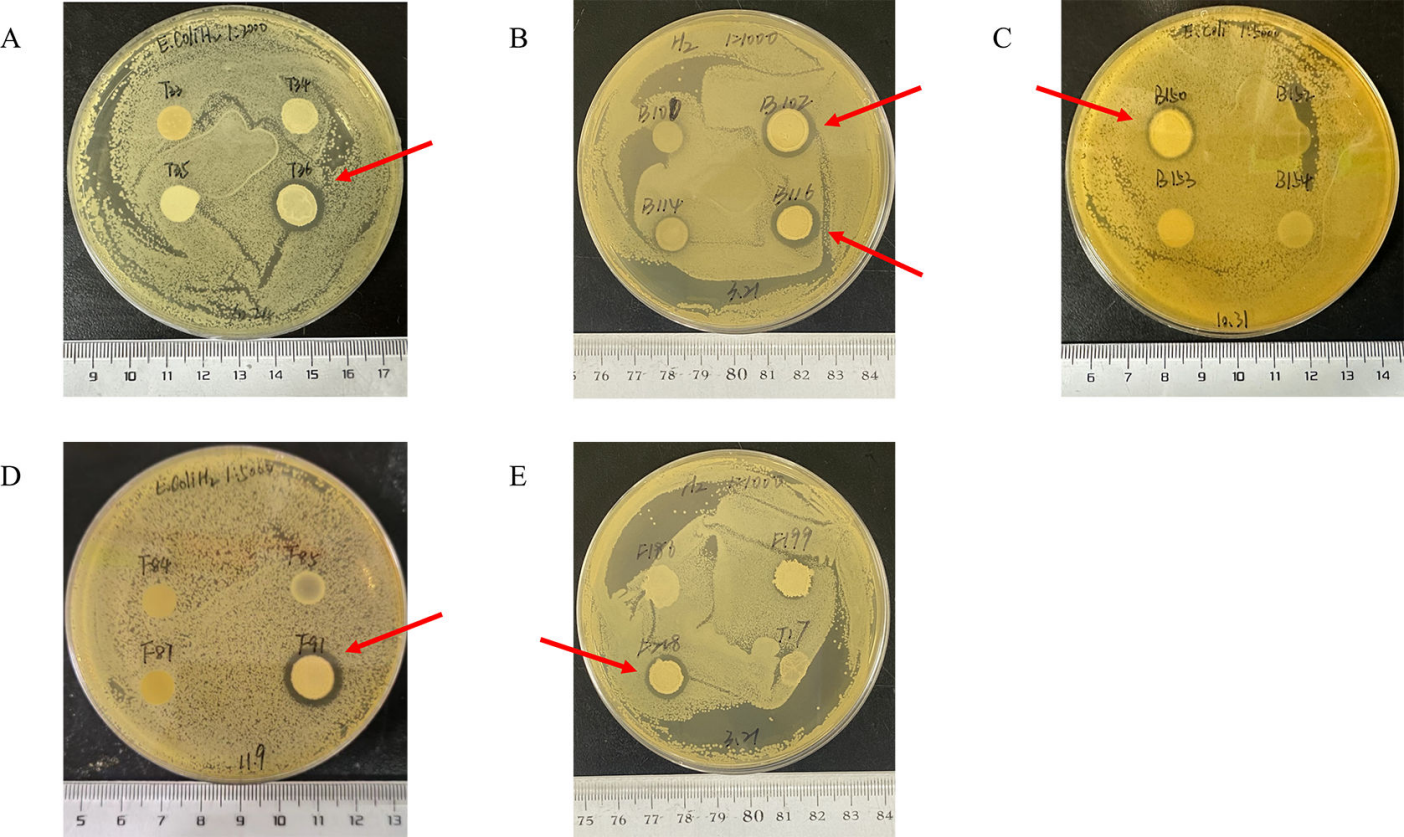
Significant antibacterial activity of six strains of *Bacillus* against *E. coli* K99. (**A**) T36. (**B**) B102 and B116. (**C**) B150. (**D**) F91. (**E**) F218.

The antimicrobial activity of the 39 *Bacillus* strains was evaluated using the hole diffusion method. The results, shown in Fig. 2, revealed that all 39 *Bacillus* strains exhibited inhibition zones greater than 9 mm in diameter against *E. coli* K99. Among them, the inhibition zones for strains T36, B102, B116, B150, B36, and F91 were 14.67 mm, 14.67 mm, 14.34 mm, 14.67 mm, 14 mm, and 14 mm, respectively (Fig. 3), indicating strong antimicrobial activity against *E. coli* K99.

### *Bacillus* strains exhibit hemolytic activity

**Fig 4:**
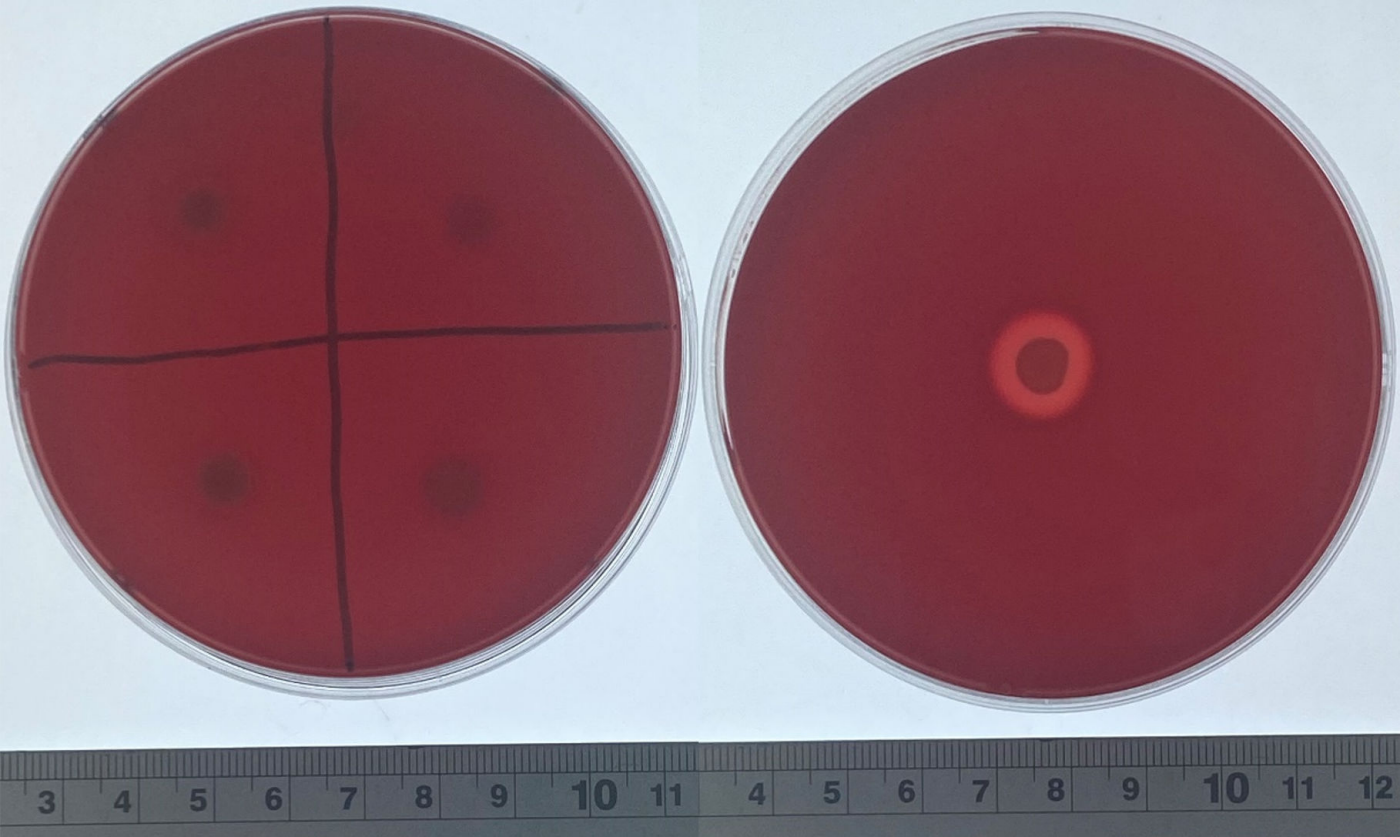
Hemolysis test: 5 µL of the culture solution was spotted onto a blood agar plate, followed by aerobic incubation at 37°C for 24 hours. The next day, the presence of a clear zone around the bacterial colonies was observed. Left: T36, B102, B116, and B150 in clockwise order starting from the upper right corner. Right: positive control (*S. aureus* CMCC(B)26003).

To further evaluate the *in vitro* safety of the *Bacillus* strains, hemolysis tests were conducted. The results (Table 3) revealed that 22 of the 39 *Bacillus* strains exhibited a hemolytic zone around their colonies, indicating hemolytic activity, while no hemolytic zones were observed around the colonies of the remaining 17 strains, suggesting that these strains do not exhibit hemolytic activity and are considered safe (Fig. 4).

**TABLE 3:** Determination of hemolytic activity of 39 strains of *Bacillus* sp.^*a*^

Strain	Hemolytic activity	Strain	Hemolytic activity
B36	β	F140	γ
B43	γ	F144	β
B53	β	F173	β
B60	β	F179	β
B72	γ	F186	γ
B91	β	F218	γ
B100	β	T36	γ
B102	γ	T37	γ
B114	β	T63	γ
B116	γ	T17	β
B137	β	T48	γ
B139	β	T56	β
B142	β	B46	β
B150	γ	B65	β
B289	β	B126	β
F17	β	F133	γ
F57	β	F149	γ
F60	γ	F165	β
F91	β	F199	γ
F108	γ		

^*a*^ “β” indicate positive reaction, and “γ” indicates negative reaction.

Based on the combined results from biofilm formation assays, antibacterial activity tests against *E. coli* K99, and hemolysis assays, the strains with strong biofilm-forming abilities, no hemolytic activity, and good antibacterial effects—T36, B102, B116, and B150—were selected for further experiments.

### T36, B102, B116, and B150 exhibit strong hydrophobicity, self-aggregation, and coaggregation abilities

Hydrophobicity, self-aggregation, and coaggregation abilities are critical parameters for evaluating the probiotic potential of *Bacillus* strains. The results (Table 4) revealed that the hydrophobicity of T36, B102, B116, and B150 was 43.85±0.94%, 44.63±3.30%, 49.61±2.41%, and 40.8±1.67%, respectively, significantly higher than that of *B. subtilis* SHBCC D50761, which was 35.15 ± 4.02%.

**TABLE 4:** Hydrophobicity, self-aggregation, and coaggregation rates of T36, B102, B116, and B150

Strain	Hydrophobicity (%)	Autoaggregation (%)	Coaggregation (%)
	Xylene	2h	*E. coli* K99
T36	43.85 ± 0.94	34.11 ± 1.44	19.64 ± 1.17
B102	49.61 ± 2.41	37.94 ± 2.22	26.31 ± 1.73
B116	44.63 ± 3.30	38.29 ± 2.22	25.67 ± 2.82
B150	40.8 ± 1.67	36.89 ± 1.59	24.66 ± 1.93
*B. subtilis* SHBCC D50761	35.15 ± 3.52	30.35 ± 1.98	16.44 ± 1.53
PBS	20 ± 1.54		

The self-aggregation rates of T36, B102, B116, and B150 were 34.11 ± 1.44%, 37.94 ± 2.22%, 38.29 ± 2.22%, and 36.89 ± 1.59%, respectively, also notably higher than *B. subtilis* SHBCC D50761, which had a self-aggregation rate of 30.35 ± 1.98%.

In terms of coaggregation with *E. coli* K99, T36, B102, B116, and B150 showed coaggregation rates of 19.64 ± 1.17%, 26.31 ± 1.73%, 25.67 ± 2.82%, and 24.66 ± 1.93%, respectively. In comparison, *B. subtilis* SHBCC D50761 exhibited a coaggregation rate of 16.44 ± 1.53% with *E. coli* K99. Compared to *B. subtilis* SHBCC D50761, T36, B102, B116, and B150 demonstrated significantly higher coaggregation capabilities.

These findings indicate that T36, B102, B116, and B150 possess excellent hydrophobicity, self-aggregation, and coaggregation abilities, highlighting their promising potential as probiotics.

### T36, B102, B116, and B150 exhibit significant acid, bile salt, and heat tolerance

**Fig 5:**
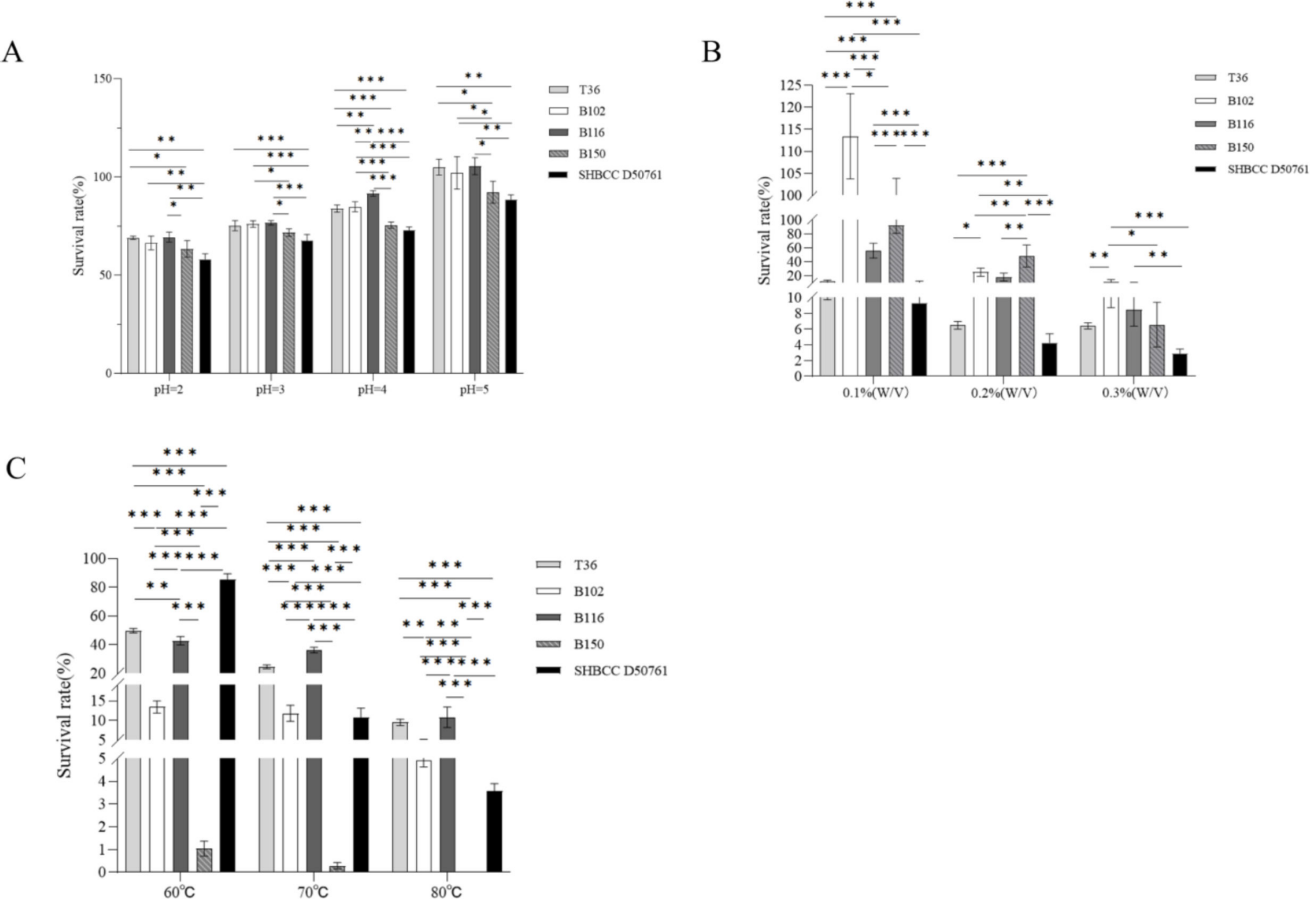
Acid, bile salt, and heat tolerance of T36, B102, B116, and B150. (**A**) Survival rate of T36, B102, B116, and B150 in BHI liquid medium at pH 2, pH 3, pH 4, and pH 5. (**B**) Survival rate of T36, B102, B116, and B150 in BHI liquid medium containing 0.1% (wt/vol), 0.2% (wt/vol), and 0.3% (wt/vol) bile salts. (**C**) Survival rate of T36, B102, B116, and B150 at different temperatures. The data represent the average of three replicates. (*P < 0.05; **P < 0.01; ***P < 0.001; ns indicates no significant difference).

The results of the acid tolerance test (Fig. 5A) show that, under pH conditions of 2, 3, 4, and 5, the survival rates of T36, B102, and B116 were significantly higher than that of *B. subtilis* SHBCC D50761. The survival rate of B150 was not significantly different from that of *B. subtilis* SHBCC D50761. In the bile salt tolerance experiment (Fig. 5B), at concentrations of 0.1% (wt/vol), 0.2% (wt/vol), and 0.3% (wt/vol) bile salts, the survival rates of B102, B116, and B150 were significantly higher than that of *B. subtilis* SHBCC D50761, while T36’s survival rate showed no significant difference compared to *B. subtilis* SHBCC D50761. The heat tolerance results (Fig. 5C) indicated that at 50°C, the survival rates of T36, B102, B116, and B150 were significantly lower than that of *B. subtilis* SHBCC D50761; however, at 60°C and 70°C, the survival rates of T36, B102, and B116 were significantly higher than that of *B. subtilis* SHBCC D50761. As the temperature increased, the survival rates of T36, B102, B116, B150, and *B. subtilis* SHBCC D50761 gradually decreased.

In conclusion, T36, B102, and B116 demonstrate significant resistance to acid, bile salts, and heat, suggesting their potential as robust strains for further applications.

### T36, B102, B116, and B150 exhibit significant resistance to gastric and intestinal fluids

**Fig 6:**
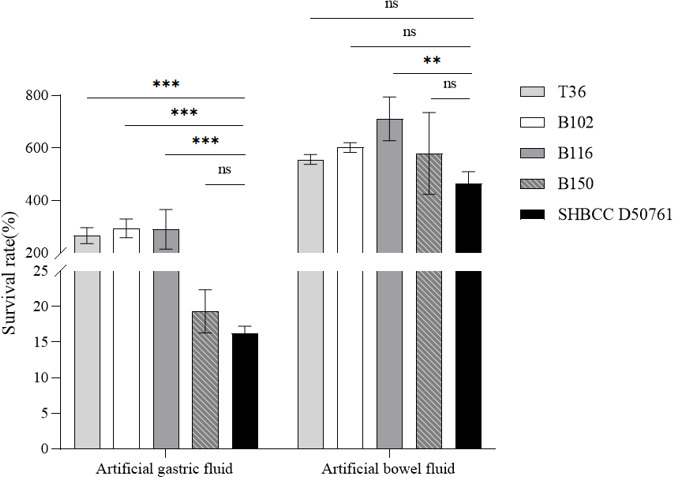
Artificial gastric fluid and artificial bowel fluid tolerance of T36, B102, B116, and B150. The overnight culture medium was diluted 1:50 (1 mL) and transferred to prepared simulated gastric and intestinal fluids, and aerobic culture was performed at 37°C for 4 hours. Bacterial viability was assessed at 0 and 4 hours using the drop plate method. The data represent the average of three replicates. (**P < 0.01; ***P < 0.001; ns indicates no significant difference).

The results (Fig. 6) showed that the survival rates of T36, B102, and B116 in artificial gastric fluid were significantly higher than that of *B. subtilis* SHBCC D50761. The survival rate of B150 in artificial gastric fluid did not differ significantly from that of *B. subtilis* SHBCC D50761. In artificial intestinal fluid, the survival rates of T36, B102, and B150 were comparable to that of *B. subtilis* SHBCC D50761, while B116 showed significantly higher survival rates than *B. subtilis* SHBCC D50761. These results indicate that T36, B102, and B116 possess strong resistance to artificial gastric digestion, while B116 demonstrates excellent resistance to artificial intestinal digestion.

### T36, B102, B116, and B150 demonstrate good *in vitro* safety profiles

**Fig 7:**
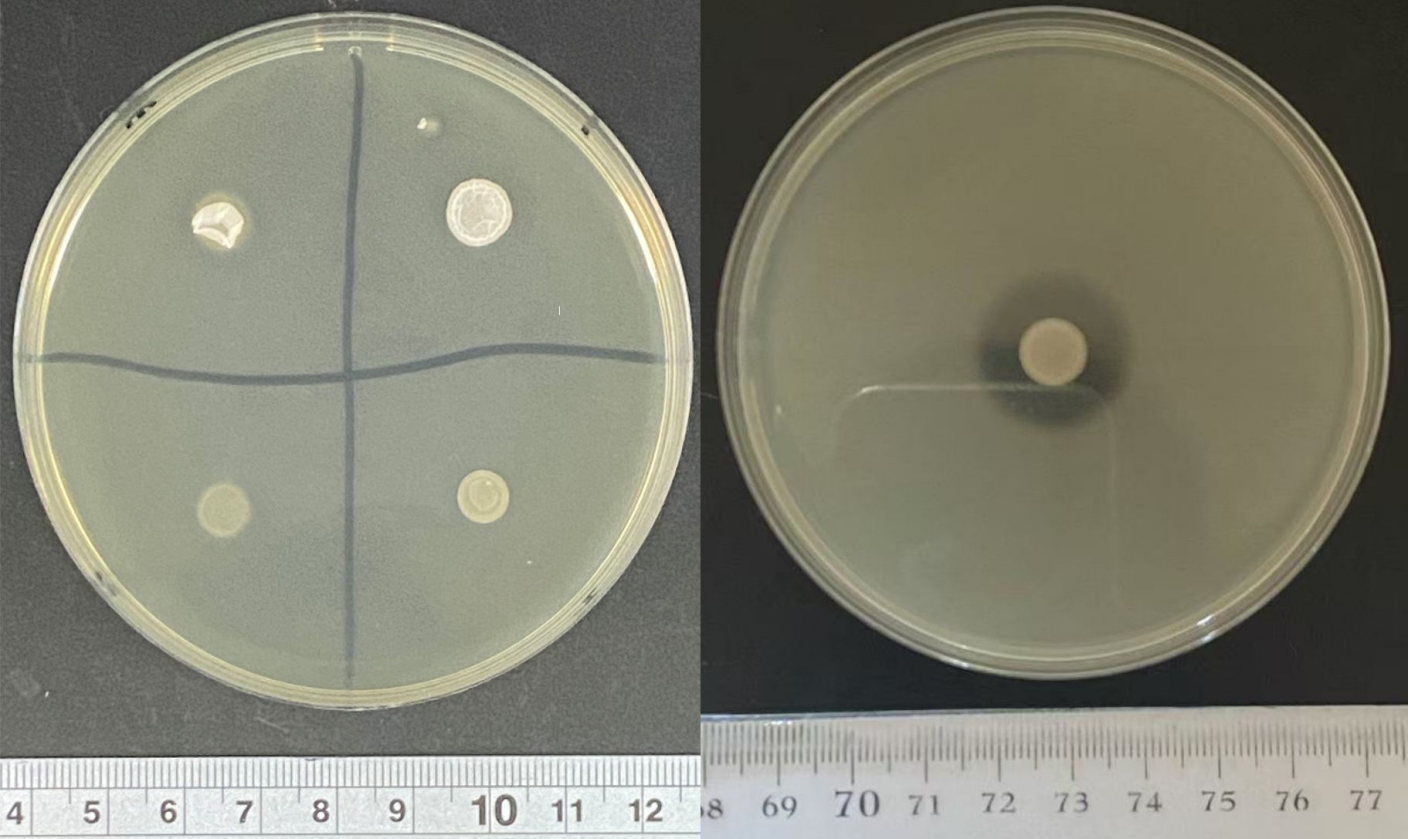
Gelatinase test: 5 µL of the overnight culture was spotted onto a gelatin agar plate, followed by aerobic incubation at 37°C for 24 hours. The formation of a clear, transparent zone around the colonies was then observed. Left: T36, B102, B116, and B150 in clockwise order starting from the upper right corner. Right: positive control (*P. aeruginosa* ATCC 9027).

**Fig 8:**
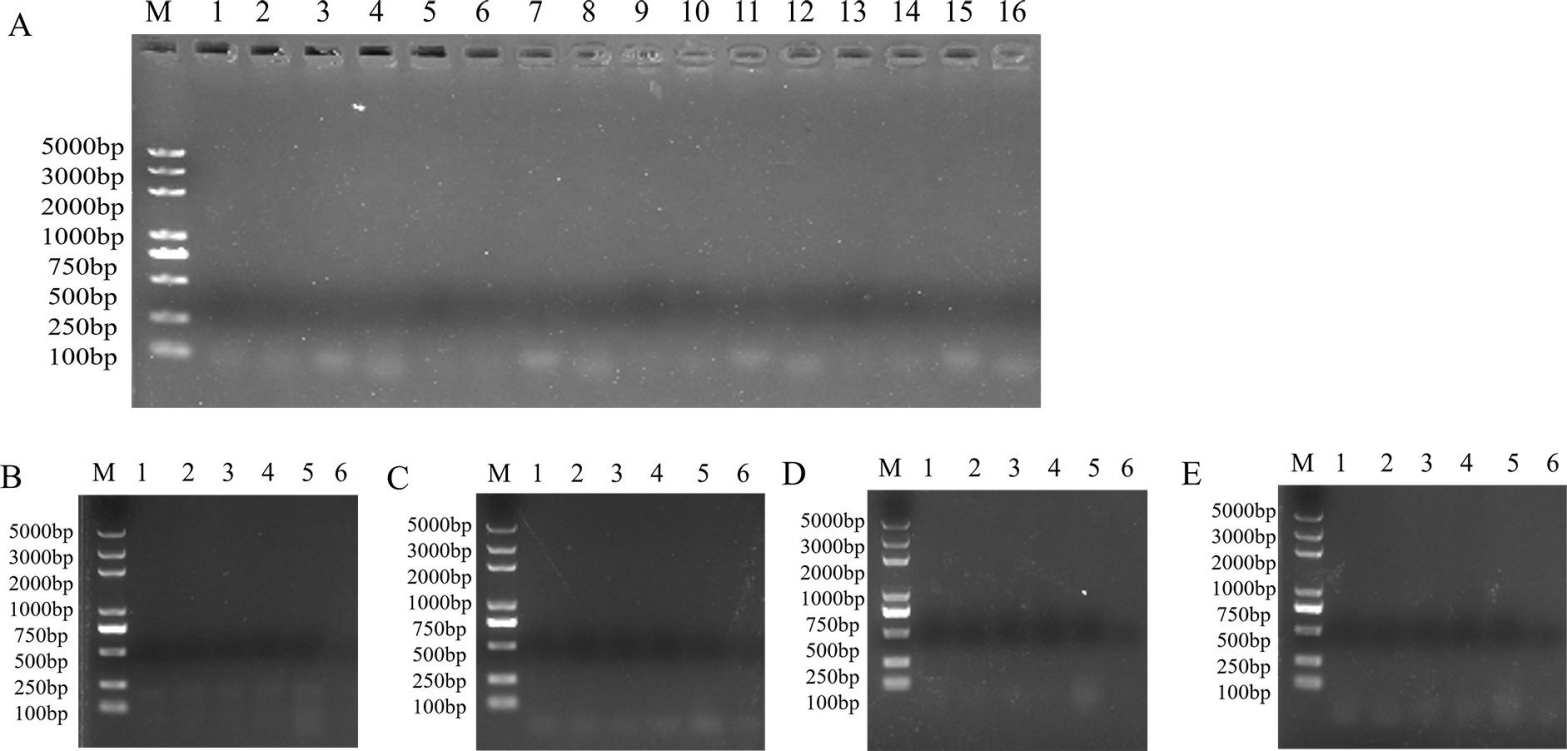
PCR detection of antibiotic resistance genes and virulence genes, and in T36, B102, B116, and B150. (**A**) Electrophoresis images of T36, B102, B116, and B150 antibiotic resistance genes. M, marker DL2000; T36 lanes 1–4 correspond to the *vanA*, *vanB*, *lnuA*, *rpoB* genes, respectively. B102, lanes 5–8. B116, lanes 9–12. B150, lanes 13–16. (**B**) Electrophoresis image of T36 virulence genes: M: marker DL2000; lanes 1–6 correspond to the *nheA, nheB, nheC, hblA, hblC,* and *hblD* genes, respectively. (**C**) Electrophoresis image of B102 virulence genes. (**D**) Electrophoresis image of B116 virulence genes. (**E**) Electrophoresis image of B150 virulence genes.

To further assess the *in vitro* safety of these strains, we evaluated their gelatinase activity, antibiotic sensitivity, and presence of virulence genes. The gelatinase test (Fig. 7) showed no clear zones around the colonies of T36, B102, B116, and B150, indicating that none of these strains produce gelatinase. The antibiotic resistance assay (Table 5) revealed that T36, B102, B116, and B150 exhibited intermediate resistance to four antibiotics—streptomycin, lincomycin, polymyxin B, and tetracycline—while they were sensitive to 20 other antibiotics, including ampicillin, gentamicin, sulfamethoxazole, and florfenicol. Virulence gene detection (Fig. 8) showed that T36, B102, B116, and B150 lacked both resistance genes and virulence genes. In conclusion, these results indicate that T36, B102, B116, and B150 possess excellent *in vitro* safety.

**TABLE 5:** Antimicrobial susceptibility profile of T36, B102, B116, and B150

Antibiotic		Drug content (μg/ tablet)	Standard inhibition circle diameter (mm)			T36	B102	B116	B150
			Sensitive (S)	Intermediate-resistant (I)	Resistant (R)				
1	Streptomycin (S)	10	≥15	12–14	≤11	I	I	I	I
2	Lincomycin (MY)	2	≥20	11–19	≤10	I	I	I	I
3	Ampicillin (AMP)	10	≥17	13–16	≤12	S	S	S	S
4	Gentamicin (GEN)	10	≥15	13–14	≤12	S	S	S	S
5	Sulfafurazole (SIZ)	30	≥16	11–15	≤10	S	S	S	S
6	Florfenicol (FON)	30	≥17	15–16	≤14	S	S	S	S
7	Kanamycin (KAN)	30	≥18	14–17	≤13	S	S	S	S
8	Polymyxin B (PB)	300	≥12	9–11	≤8	I	I	I	I
9	Ceftriaxone (CRO)	30	≥21	14–20	≤13	S	S	S	S
10	Ceftazidime (CAZ)	30	≥18	15–17	≤14	S	S	S	S
11	Tetracycline (TET)	30	≥19	15–18	≤14	I	I	I	I
12	Ciprofloxacin (CIP)	5	≥26	22–25	≤21	S	S	S	S
13	Penicillin (PEN)	10	≥23	20–22	≤19	S	S	S	S
14	Cefoperazone (CPZ)	75	≥21	16–20	≤15	S	S	S	S
15	Erythromycin (E)	15	≥23	14–22	≤13	S	S	S	S
16	Vancomycin (VAN)	30	≥12	10–11	≤9	S	S	S	S
17	Chloromycetin (C)	30	≥18	13–17	≤12	S	S	S	S
18	Neomycin (N)	30	≥19	15–18	≤14	S	S	S	S
19	Azithromycin (AZM)	15	≥16	11–15	≤10	S	S	S	S
20	Ceftazidime (CAZ)	30	21	15–17	17	S	S	S	S
21	Trimethoprim (TMP)	5	16	11–15	10	S	S	S	S
22	Enrofloxacin (ENR)	30	26	22–25	21	S	S	S	S
23	Oxacillin (OX)	30	≥23	20–22	≤19	S	S	S	S
24	Clindamycin (CC)	30	≥23	14–22	≤13	S	S	S	S

Based on the combined results from hydrophobicity, self-aggregation, and coaggregation tests, acid tolerance, bile salt tolerance, heat resistance, artificial gastric and intestinal fluid tolerance, and *in vitro* safety assessments, we selected B102 and B116 for further *in vivo* safety and probiotic efficacy testing.

### B102 and B116 exhibit favorable *in vivo* safety characteristics

Gastric gavage in mice is a crucial method for assessing the safety of probiotics *in vivo*. In this study, B102 and B116 were administered via gavage at the same doses, followed by acute bacterial challenge and continuous-gavage experiments.

#### Acute bacterial challenge experiment

The results of the acute bacterial challenge experiment demonstrated that after a 14-day gavage of the highest dose (2 × 10⁹ CFU per mouse) of B102 or B116, no mortality was observed in the mice. Throughout the gavage period, the mice’s visual, digestive, nervous, and respiratory systems, as well as other organ systems, remained normal (Table 6). These findings indicate that B102 and B116 do not cause acute toxicity in mice.

**TABLE 6:** Clinical manifestations of mice undergoing acute bacterial challenge with B102 and B116

Strain	Organ/System	Trait manifestation
B102	Eye	Pupil normal, without dilation or constriction
		Eyeball normal, without protrusion
		Eyelid normal
		Periorbital secretions no tearing
	Skin and mucosa	Mucosa no mucus discharge
		Oral cavity normal
	Hair coat	Color normal
		Integrity not loose
	Secretions and excretions	Feces formed, normal color
		Abdominal shape no diarrhea or constipation
		Nostril normal, no runny nose
	Nervous system	Normal body position, normal vocalization, no abnormal postures
	Behavioral manifestations	No presence of tonic or clonic activities
		No presence of abnormal behaviors
		Normal stress response
B116	Eye	Pupil normal, without dilation or constriction;
		Eyeball normal, without protrusion
		Eyelid normal
		Periorbital secretions no tearing
	Skin and mucosa	Mucosa no mucus discharge
		Oral cavity normal
	Hair coat	Color normal
		Integrity not loose
	Secretions and excretions	Feces formed, normal color
		Abdominal shape no diarrhea or constipation
		Nostril normal, no runny nose
	Nervous system	Normal body position, normal vocalization, no abnormal postures
	Behavioral manifestations	No presence of tonic or clonic activities
		No presence of abnormal behaviors
		Normal stress response

#### 28-Day oral toxicity test

##### BMI measurement

**Fig 9:**
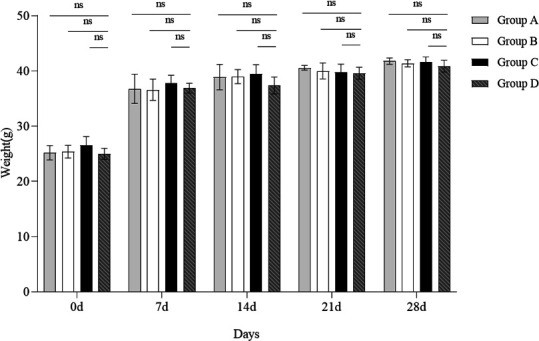
Body mass index of mice after administration of B102, B116, and *B. subtilis* SHBCC D50761. The data represent the average of three replicates. (ns means not significant difference).

The results of the BMI measurement (Fig. 9) indicated that there were no significant differences in BMI between the gavaged groups (B102 [group A], B116 [group B], and *B. subtilis* SHBCC D50761 [group C]) and the control group (group D). This suggests that gavage with different *Bacillus* strains does not lead to significant changes in body weight in mice.

##### Organ index measurement

The organ index measurements (Table 7) showed no significant differences in the heart-to-body ratio, liver-to-body ratio, spleen-to-body ratio, lung-to-body ratio, or kidney-to-body ratio between the gavaged groups (groups A, B, and C) and the control group (group D). These results indicate that gavage with B102, B116, or *B. subtilis* SHBCC D50761 does not affect the organ weights of mice.

**TABLE 7:** Organ index of mice administered B102, B116, and *B. subtilis* SHBCC D50761

Group	Heart-to-body ratio/%	Liver-to-body ratio/%	Spleen-to-body/%	Lung-to-body ratio/%	Kidney-to-body ratio/%
A	0.36 ± 0.05	3.89 ± 0.44	0.22 ± 0.08	0.75 ± 0.16	0.90 ± 0.09
B	0.34 ± 0.01	4.25 ± 0.34	0.23 ± 0.02	0.72 ± 0.20	1.00 ± 0.14
C	0.35 ± 0.05	3.96 ± 0.28	0.22 ± 0.04	0.76 ± 0.18	0.96 ± 0.06
D	0.31 ± 0.06	3.95 ± 0.21	0.21 ± 0.07	0.72 ± 0.10	0.91 ± 0.05

##### Bacterial translocation experiment

The results from the bacterial translocation experiment demonstrated that after scraping internal tissues from the organs of mice in the gavaged groups (groups A, B, and C) and the control group (group D), no bacterial colonies were detected in any of the samples. This suggests that gavage of B102, B116, or *B. subtilis* SHBCC D50761 does not cause bacterial translocation or infection in other organs of the mice.

##### Blood biochemical analysis

Blood biochemical markers play a vital role in evaluating the safety of probiotics. As shown in Table 8, there were no significant differences in platelet count (PLT), red blood cell count (RBC), white blood cell count (WBC), hemoglobin content (HGB), mean corpuscular volume (MCV), or mean corpuscular hemoglobin (MCH) between the gavage groups (groups A, B, and C) and the control group (group D). These findings suggest that B102, B116, and *B. subtilis* SHBCC D50761 do not have a significant impact on the hematological parameters of mice.

**TABLE 8:** Blood biochemical indices in mice after administration of B102, B116, and *B. subtilis* SHBCC D50761

Group	PLT (10^9^/L)	RBC (10^12^/L)	WBC (10^9^/L)	HGB (g/L)	MCV (fL)	MCH (pg)
A	479 ± 40.79	9 ± 0.82	2 ± 0.70	152 ± 6.20	59 ± 4.89	18 ± 1.13
B	497 ± 53.43	9 ± 0.68	2 ± 0.60	158 ± 5.83	60 ± 3.87	17 ± 1.64
C	406 ± 89.15	9 ± 0.50	2 ± 0.90	161 ± 7.99	62 ± 2.90	17 ± 0.55
D	415 ± 44.12	9 ± 0.79	2 ± 0.93	161 ± 6.99	63 ± 3.60	18 ± 1.85

**Fig 10:**
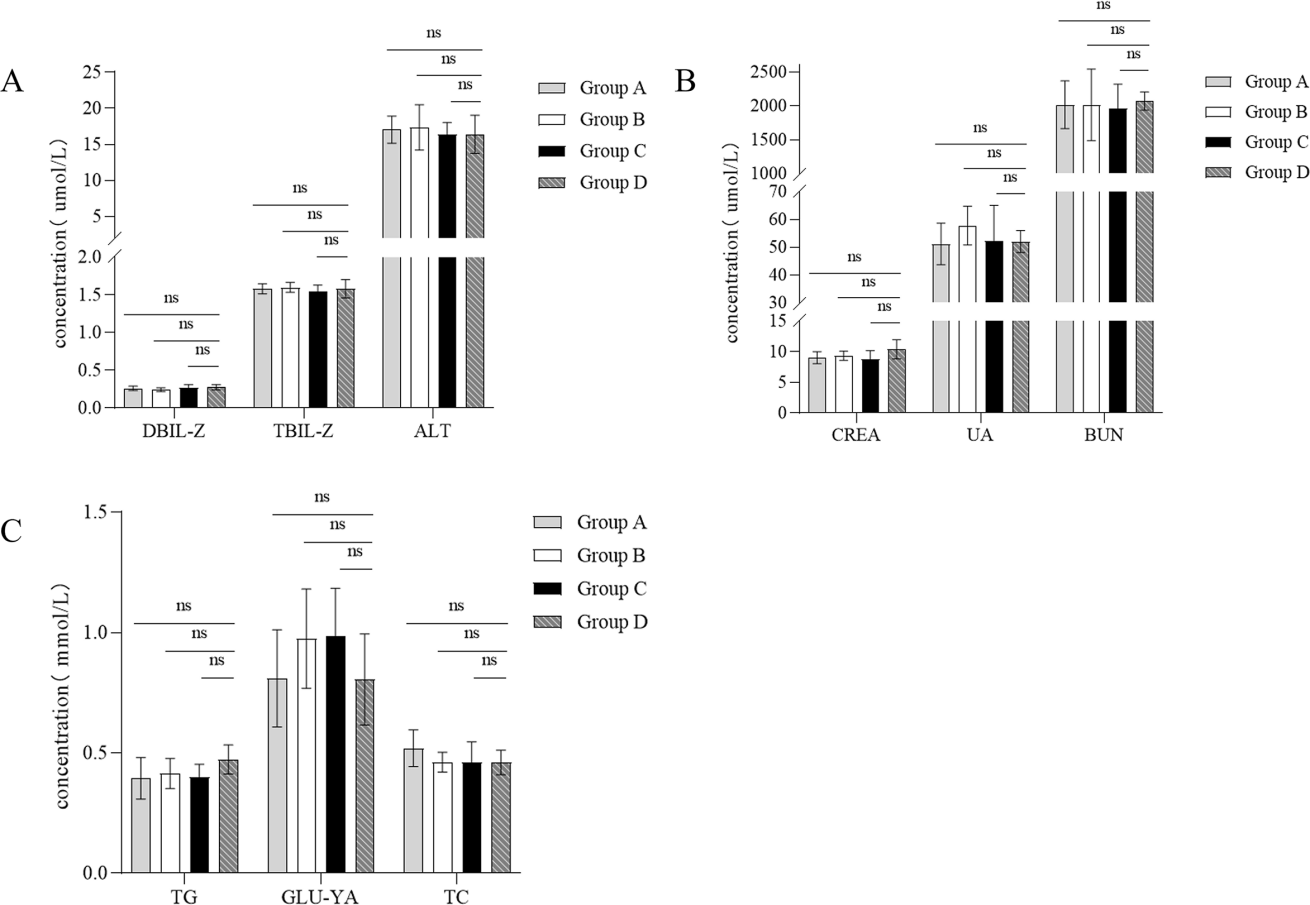
Serum biochemical analysis of mice administered B102, B116, and SHBCC D50761 via gavage. (**A**) Liver function markers: DBIL-Z represents direct bilirubin; TBIL-Z represents total bilirubin; ALT represents alanine aminotransferase. (**B**) Kidney function markers: CREA represents creatinine; UA represents uric acid; BUN represents blood urea nitrogen. (**C**) Blood glucose, lipid, and cholesterol markers: TG represents triglycerides; GLU-YA represents glucose; TC represents total cholesterol. The data represent the average of three replicates. (ns means not significant difference).

Figure 10 shows that, compared to the control group (group D), there were no significant changes in the concentrations of direct bilirubin, total bilirubin, and alanine aminotransferase in the gavaged groups (groups A, B, and C). These results indicate that B102, B116, and *B. subtilis* SHBCC D50761 do not impair liver cell integrity or liver function in mice. Similarly, there were no significant changes in the concentrations of blood urea nitrogen, uric acid, and creatinine in the gavaged groups (groups A, B, and C) compared to the control group (group D), indicating that these strains do not affect nitrogen metabolism or renal excretion function. Furthermore, there were no significant changes in the levels of total cholesterol, glucose, and triglycerides in the gavaged groups, suggesting that B102, B116, and *B. subtilis* SHBCC D50761 do not cause adverse effects on blood sugar or lipid metabolism in mice.

##### Histological examination of the ileum

**Fig 11:**
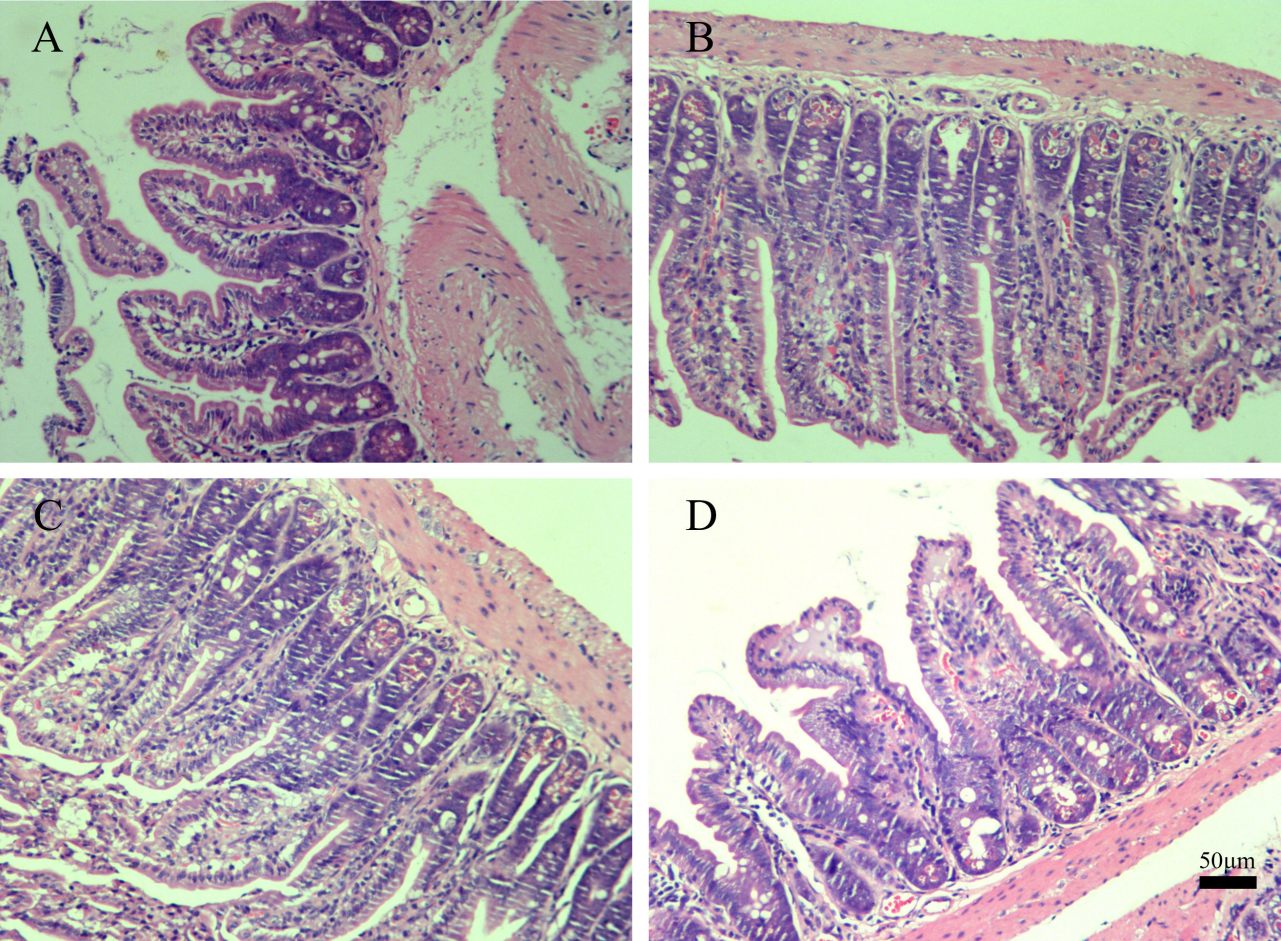
Histopathological examination of the ileum in mice administered B102 and B116 via gavage (200×). (**A**) Group A: mice gavaged with B102 at a dose of 2 × 10^7^ CFU per mouse. (**B**) Group B: mice gavaged with B116 at a dose of 2 × 10^7^ CFU per mouse. (**C**) Group C: mice gavaged with *B. subtilis* SHBCC D50761 at a dose of 2 × 10^7^ CFU per mouse. (**D**) Group D: mice gavaged with 200 µL of PBS.

The histological examination of the ileum was conducted to evaluate the impact of B102, B116, and *B. subtilis* SHBCC D50761 on the integrity of the intestinal mucosa. The results (Fig. 11) showed no significant changes in the intestinal morphology of the ileum in the gavaged groups (groups A, B, and C) compared to the control group (group D), and no pathological findings were observed in any of the groups. These findings indicate that B102, B116, and *B. subtilis* SHBCC D50761 do not cause damage to the ileum in mice.

In conclusion, the results from the *in vivo* safety assessments suggest that B102, B116, and *B. subtilis* SHBCC D50761 exhibit a favorable safety profile.

### B102 and B116 provide protective effects against *E. coli* K99 infection in mice

**Fig 12:**
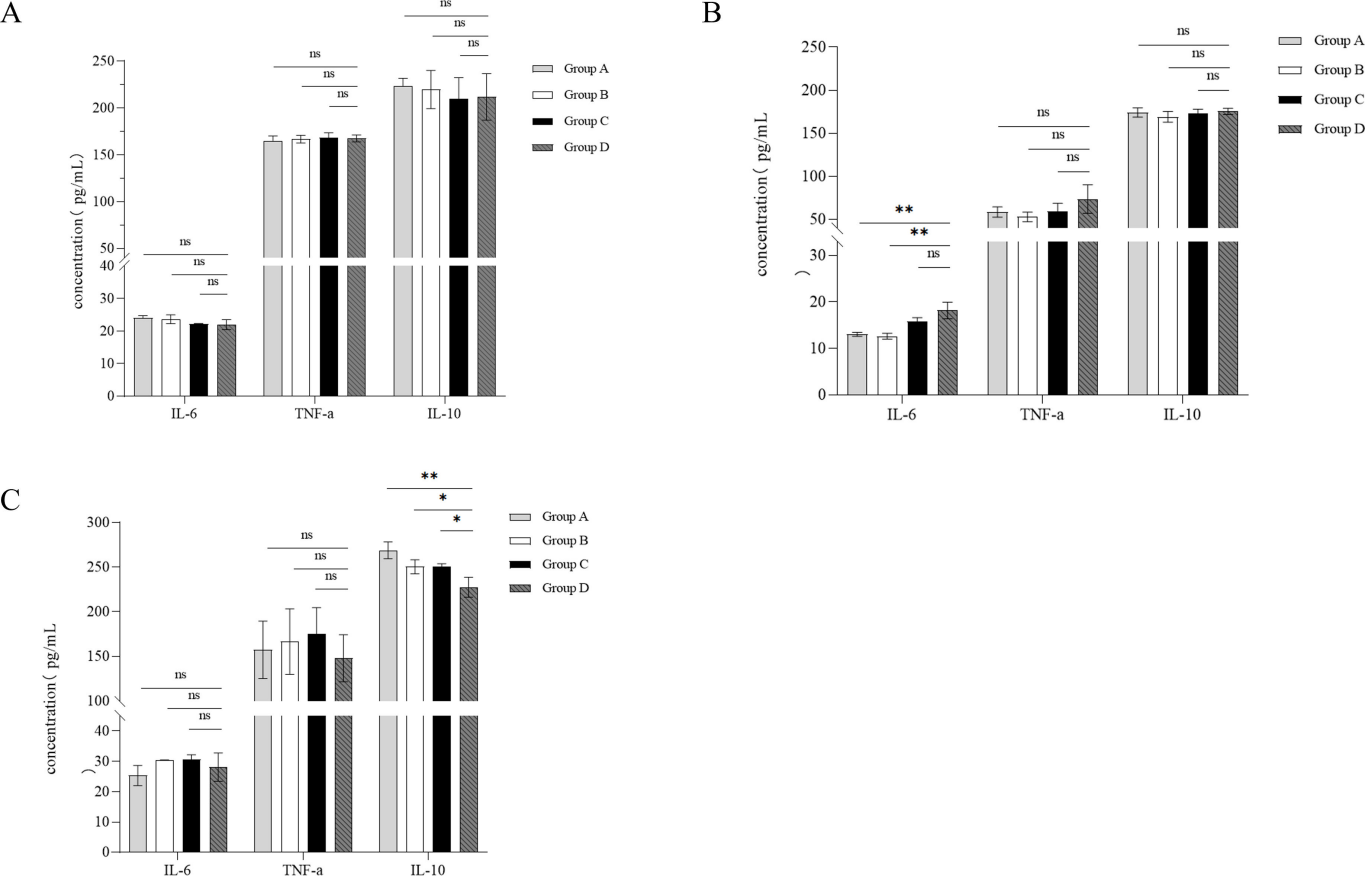
Tissue samples (0.1 g) were homogenized in 1 mL of PBS, centrifuged at 3,000 rpm for 20 min at 4°C, and the supernatants were stored at −80°C. The expression levels of IL-6, TNF-α, and IL-10 cytokines in different mouse tissues after oral administration of B102, B116, and *Bacillus subtilis* SHBCC D50761 were determined using mouse ELISA kits (Jiangsu Su-Lian Ke Biotechnology Co., Ltd.). (**A**) Serum. (**B**) Jejunum. (**C**) Colon. The data represent the average of three replicates. (*P < 0.05; **P < 0.01; ns means not significant difference).

To evaluate whether B102 and B116 could mitigate the inflammatory responses caused by *E. coli* K99, this study measured the levels of inflammatory cytokines TNF-α, IL-6, and IL-10 in the serum, jejunal, and colonic tissues. The results showed that, compared to the control group (group D), there were no significant changes in the levels of IL-6, TNF-α, and IL-10 in the serum of the gavaged groups (groups A, B, and C) (Fig. 12A). In the jejunal tissue, the expression levels of TNF-α and IL-10 did not differ significantly between the gavaged groups (groups A, B, and C) and the control group (group D). However, the IL-6 expression levels in groups A and B were significantly lower than in the control group (group D) (Fig. 12B). These results suggest that gavage administration of B102 and B116 significantly reduced the concentration of the pro-inflammatory cytokine IL-6 in the jejunum.

In the colon, there were no significant differences in the expression levels of IL-6 and TNF-α between the gavaged groups (A, B, and C) and the control group (group D), but the expression level of IL-10 in the gavaged groups was significantly higher than in the control group (Fig. 12C). These findings indicate that gavage administration of B102, B116, and *B. subtilis* SHBCC D50761 significantly increased the concentration of the anti-inflammatory cytokine IL-10 in the colon.

**Fig 13:**
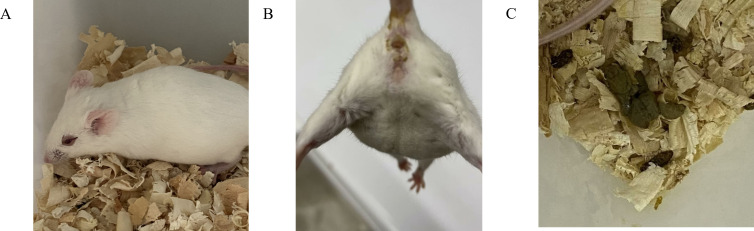
Clinical symptoms of mice infected with *E. coli* K99. (**A**) Swelling of the mouse eyelids with increased secretion. (**B**) Fecal contamination around the mouse anus. (**C**) Gray-green, pasty, loose stool excreted by the mouse.

To assess the protective effect of B102, B116, and *B. subtilis* SHBCC D50761 against *E. coli* K99 infection, the median lethal dose (LD_50_) of *E. coli* K99 was first determined. Mice were infected with *E. coli* K99 via intraperitoneal injection and exhibited symptoms such as tremors, an arched back, swollen eyelids with increased secretion, fecal contamination around the anus, and the expulsion of gray-green, pasty diarrhea (Fig. 13). Mortality was observed starting on day 1. The LD_50_ calculation indicated that the LD_50_ of *E. coli* K99 was 4.4 × 10^7^ CFU per mouse (Table 9).

**TABLE 9:** LD_50_ of *E. coli* K99 in KM mice

Strain	Inoculation dose (CFU per mouse)	No. of deaths/total no. of mice	LD_50_ (CFU per mouse)
*E. coli* K99	5.56 × 10^8^	5/5	4.4 × 10^7^
	5.56 × 10^7^	3/5	
	5.56 × 10^6^	0/5	
	5.56 × 10^5^	0/5	
	5.56 × 10^4^	0/5	

**Fig 14:**
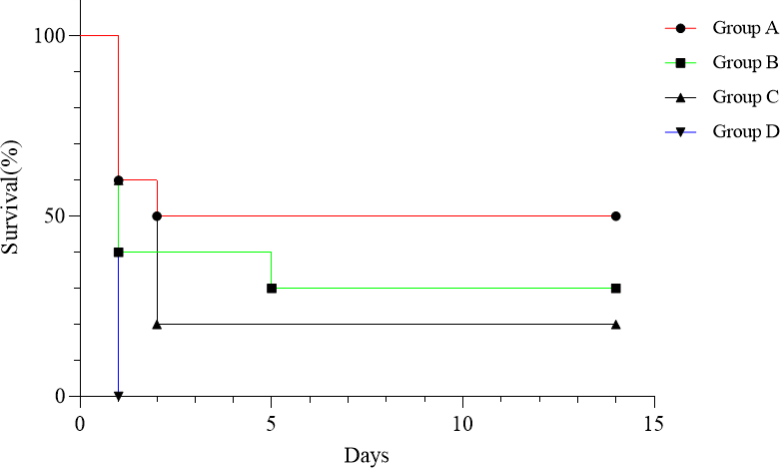
Survival rate after intraperitoneal challenge with *E. coli* K99 following oral administration of *Bacillus*.

To further assess the protective effects of strains B102, B116, and *B. subtilis* SHBCC D50761, mice were administered these strains via oral gavage for 14 consecutive days, followed by intraperitoneal injection with *E. coli* K99. The incidence and severity of diarrhea were then monitored. As shown in Table 10, mice in the treatment groups (groups A, B, and C) exhibited normal behavior, prompt responsiveness, no periorbital secretions, normal feeding and drinking habits, and well-formed, elliptical, dark brown feces with regular defecation patterns. In contrast, mice in the control group (group D) displayed typical signs of diarrhea, including reduced activity, huddling in the corners of the cage, sluggish responses to external stimuli, swollen eyelids with excessive secretions, markedly decreased food intake, loose and unshaped feces with a gray-green coloration, increased defecation frequency, and perianal contamination with fecal matter. In the meantime, the survival rate was recorded to generate a survival curve. The results (Fig. 14) showed that the survival rate in group A was 50%, in group B was 30%, in group C was 20%, and in group D was 0%. The survival rate in group A was significantly higher than in group D. These findings indicate that gavage administration of B102 and B116 significantly reduces mortality caused by *E. coli* K99 infection in mice, with noticeable improvements in diarrhea symptoms.

**TABLE 10:** The clinical manifestations of diarrhea in mice

Clinical inspection	Group A	Group B	Group C	Group D
Actions and behaviors	Rapid response	Rapid response	Rapid response	Lethargy, huddling
Visual system	Normal eyelids and no discharge	Normal eyelids and no discharge	Normal eyelids and no discharge	Swollen eyelids, excessive discharge
Digestive system	Normal	Normal	Normal	Reduced feed intake
Feces	Feces formed, normal color	Feces formed, normal color	Feces formed, normal color	Grayish-green, unformed stools
Defecation frequency	Normal	Normal	Normal	Increased
Changes around the anus	No perianal fecal staining	No perianal fecal staining	No perianal fecal staining	Perianal fecal staining

## DISCUSSION

*E. coli* K99 is one of the most common bacteria responsible for bacterial diarrhea in young ruminants (22, 23). It causes severe watery diarrhea and dehydration, which can lead to death. Probiotics have gained significant attention for their potential in preventing pathogenic infections. A study by Wu et al. demonstrated that a complex of *Lactobacillus acidophilus*, *B. subtilis*, and *Saccharomyces cerevisiae* effectively inhibited the intestinal colonization of *E. coli* K99 by directly or indirectly antagonizing bacterial adhesion to epithelial cells. In this study, 39 strains of *Bacillus* were isolated from fresh feces of healthy cattle. Four strains with strong antimicrobial activity against *E. coli* K99 and no hemolytic activity were selected, namely T36, B102, B116, and B150. Probiotic properties are strain-specific, and their capabilities depend largely on the source of the strain and its intended target. Therefore, to consider a microorganism as a potential probiotic, *in vitro* and *in vivo* properties must be thoroughly evaluated (19, 24, 25). The results of this study showed that B102 and B116 possess strong antimicrobial activity along with excellent hydrophobicity, self-aggregation, coaggregation, acid tolerance, bile salt tolerance, and heat resistance. Additionally, B102 and B116 do not exhibit gelatinase activity and demonstrate good tolerance to artificial gastrointestinal fluids. B102 and B116 did not cause acute toxicity or clinical infections in mice. Furthermore, they significantly reduced the mortality rate in a pathogenic *E. coli* K99 challenge model. These findings suggest that B102 and B116 exhibit good safety and probiotic potential in mice and could be further developed as probiotic products to prevent *E. coli*-induced diarrhea in animals.

Previous studies have highlighted the resilience of *Bacillus* spp., which are known for their ability to withstand harsh external conditions and maintain their activity under extreme environments, including high temperatures, acidity, or alkalinity (26). It is well-established that the pH of stomach acid typically ranges around 3.0, while bile concentrations in the small intestine vary between 0.1% and 0.3%. This suggests that probiotics must endure not only the low pH in the stomach but also the high bile salt concentration in the small intestine to survive and colonize the gastrointestinal tract (27). In our study, we observed that both B102 and B116 exhibited exceptional resistance to acid and bile salts, outperforming *B. subtilis* SHBCC D50761. Furthermore, B102 and B116 demonstrated superior resistance to artificial gastric fluids, with B116 showing a significantly higher tolerance than *B. subtilis* SHBCC D50761. Additionally, their resistance to artificial intestinal fluids was comparable to that of *B. subtilis* SHBCC D50761.

The autoaggregation and surface hydrophobicity of probiotics are crucial for their adhesion to intestinal epithelial cells. Coaggregation with pathogens reflects the antimicrobial capabilities of probiotics and inhibits the attachment of harmful microbes to the intestinal mucosa (28). Our results show that T36, B102, B116, and B150 had higher hydrophobicity, autoaggregation, and coaggregation rates compared to *B. subtilis* SHBCC D50761, indicating their potential for effective intestinal colonization and inhibition of pathogen adhesion.

Pathogenicity is another key factor in the safety evaluation of probiotics. The presence of hemolytic, lecithinase, and gelatinase activities is commonly associated with the ability to disrupt host cells and connective tissues. Hemolytic bacteria produce hemolysins that break down erythrocytes, which can lead to sepsis. Gelatinase, on the other hand, hydrolyzes connective tissue components, promoting pathogen invasion and contributing to intestinal diseases (29). Hence, screening for these activities is essential for ensuring the safety of potential probiotics. In this study, we tested the hemolytic activity of T36, B102, B116, and B150 using the spot inoculation method and found no hemolytic zones, confirming that these strains do not produce hemolysins.

Additionally, we examined the lecithinase activity of these strains and found no evidence of lecithinase activity in any of the four strains. These findings are consistent with previous studies, such as those by Ahire et al., who assessed the safety of *Bacillus clausii* UBBC07 (30), a spore-forming probiotic, and Iryna et al., who examined *B. clausii* UBBC07, *B. subtilis* VKPM B2335, and *Bacillus licheniformis* VKPM B2336, all of which lacked lecithinase activity (31). Gelatinase activity was also absent in our strains, as indicated by the lack of hydrolyzed areas on gelatin agar plates treated with saturated ammonium sulfate solution.

Enterotoxins, such as hemolysin BL (Hbl) and non-hemolytic enterotoxin (Nhe) (32), are significant contributors to food poisoning and diarrhea, particularly in *B. cereus* infections (33). Therefore, one of the key safety criteria for *Bacillus* strains is the absence of enterotoxin-producing genes. Our PCR analysis revealed that T36, B102, B116, and B150 did not carry the genes for Hbl (*hblA*, *hblD*, *hblC*) or Nhe (*nheA*, *nheB*, *nheC*), further confirming their safety for use as probiotics.

To assess the risk of antibiotic resistance transfer, we conducted an antibiotic susceptibility test. Our results showed that T36, B102, B116, and B150 were sensitive to 20 commonly used antibiotics, including ampicillin, gentamicin, sulfamethoxazole, florfenicol, kanamycin, ceftriaxone, ceftazidime, ciprofloxacin, penicillin, cefoperazone, erythromycin, vancomycin, chloramphenicol, azithromycin, cefotaxime, trimethoprim, enrofloxacin, benzylpenicillin, clindamycin, and neomycin. Additionally, PCR analysis did not detect any antibiotic resistance genes, including vancomycin (*vanA*, *vanB*), lincosamide (*lnuA*), or rifamycin (*rpoB*) resistance genes. These results corroborate the findings of previous studies involving *B. subtilis* TPS4, *B. velezensis* TPS3N, and *Bacillus amyloliquefaciens* TPS17, all of which were found to be sensitive to antibiotics (34, 35).

After pre-screening, two strains (B102 and B116) with superior probiotic properties were selected for animal experiments. We performed an *in vivo* safety evaluation using a mouse model. Our findings showed no acute toxicity, clinical infections, or adverse effects on growth in mice after oral administration of B102 and B116. Furthermore, blood and serum biochemical parameters, which reflect host health, showed no significant changes following administration of the probiotics. These results align with previous studies involving other probiotic strains, such as *Bacillus coagulans* and *B. licheniformis*, which did not significantly affect serum biochemical indicators in chicks (36). Histopathological analysis of the ileum tissues from mice treated with B102 and B116 revealed no signs of ulcers, inflammatory cell infiltration, or mucosal degeneration, further indicating the probiotics’ safety and their potential to promote intestinal health (37).

To further evaluate the *in vivo* efficacy of these probiotics, we orally administered B102 and B116 to mice for 14 days and then challenged the mice with *E. coli* K99. Following the infection, *E. coli* K99 releases enterotoxins, such as heat-stable enterotoxin (ST), which enter systemic circulation and cause widespread inflammation (38). Cytokines play an important role in regulating gut immunity, and intestinal inflammation often leads to a significant increase in pro-inflammatory cytokines (39). Our study showed that B102 and B116 did not induce a significant systemic inflammatory response. Notably, mice treated with B102 and B116 exhibited significantly higher levels of IL-10 expression in the colon and significantly lower levels of IL-6 in the jejunum compared to the control group. As IL-10 is a cytokine that inhibits inflammation and promotes the production of anti-inflammatory cytokines such as IL-2 and TNF-α, these results suggest that B102 and B116 may help reduce inflammation caused by bacterial infections in the gut. The study by Hu et al. (40) demonstrated that *B. subtilis* H28 significantly reduced the secretion of TNF-α, IL-1β, and IL-6, thereby alleviating host damage caused by pathogenic bacteria. Whether strains B102 and B116 exert their protective effects through modulation of cytokine levels, including inflammatory mediators, warrants further investigation. On the other hand, research by Ye et al. (41) showed that *B. velezensis* FZB42 produces antimicrobial secondary metabolites, including antibiotic lipopeptides, polyketides, and peptides. So, we hypothesize that B102 and B116 may suppress pathogens by synthesizing antimicrobial secondary metabolites, such as bacteriocins. In our future work, we plan to perform genomic sequencing of B102 and B116 to identify potential secondary metabolites and experimentally confirm their antimicrobial activity.

The survival rate of mice treated with B102 after the *E. coli* K99 challenge was 50%, while those treated with B116 had a survival rate of 30%, both significantly higher than the positive control group (*B. subtilis* SHBCC D50761), which had a survival rate of 20%. Moreover, the symptoms of diarrhea were significantly improved in these groups. Similar findings were observed in neonatal piglets treated with *Lactobacillus plantarum* following a challenge with enterotoxigenic *E. coli* K88, where probiotic treatment alleviated diarrhea and reduced mortality (24).

In conclusion, B102 and B116 demonstrated strong anti-*E*. *coli* K99 activity, favorable surface properties, and the absence of hemolytic, gelatinase, and lecithinase activities. Oral administration of B102 and B116 did not induce acute toxicity or clinical infections in mice and significantly improved survival in *E. coli* K99-challenged mice. Both strains exhibited excellent safety and probiotic characteristics both *in vitro* and *in vivo*. However, it is important to note that this study was conducted under laboratory conditions using a mouse diarrhea model. To further evaluate the application potential of B102 and B116, additional studies should assess their protective effects in large livestock, such as cattle and sheep, in farm environments against *E. coli* infections.

## Data Availability

The data that support the findings of this study are available from the corresponding author upon reasonable request.
